# Which Patients Benefit Most from Intensive Treatment in a Day Hospital for Children and Adolescents?

**DOI:** 10.1192/j.eurpsy.2025.1127

**Published:** 2025-08-26

**Authors:** I. Ezquiaga Bravo, A. Vilar Garcés, C. Pérez Lucero, N. Sampayo, K. M. Malone, I. Tomás, C. R. Santana, M. Biempica, L. M. Martín, S. Batlle Vila

**Affiliations:** Institut de Salut Mental (ISM), Hospital del Mar, Barcelona, Spain

## Abstract

**Introduction:**

Day hospitals for children and adolescents (DHCA) have experienced a notable increase in demand in the past few years, requiring an exploration of factors that predict treatment outcomes.

**Objectives:**

This study aims to investigate whether all patients derive equal benefit from intensive treatment in a day hospital or if diagnostic orientation and pre-admission severity influence therapeutic efficacy.

**Methods:**

A retrospective pre-post admission study was conducted involving adolescents treated at the DHCA Litoral Mar in Barcelona between January 2022 and December 2023. The analysis focused on the association between clinical and functional improvement, as measured by the CGAS and HoNOSCA scales, and diagnostic orientation and clinical severity, indicated by the history of suicide attempts and prior admissions. Non-parametric tests, specifically Spearman’s correlation coefficient and the Kruskal-Wallis test, were used.

**Results:**

The study sample comprised 64 patients, with a mean age of 17.72 years (SD 1.39) and 70.3% female representation. The average length of stay was 130.64 days (SD 43.92). While no statistically significant differences in clinical improvement were observed across diagnoses, patients with psychotic disorders demonstrated the most substantial improvement, followed by those with behavioral disorders, emotional regulation difficulties, and mood disorders. More modest improvements were noted for anxiety disorders and autism spectrum disorders (ASD). A statistically significant positive correlation was identified between the number of previous suicide attempts and improvement on the HoNOSCA scale (p=0.002), with no significant correlation found with previous admissions.

**Conclusions:**

The findings indicate that severe patients benefit most from intensive treatment in day hospitals, while a specific diagnosis is not a clear predictor for outcomes, probably due to the small sample size. These results underscore the importance of considering clinical severity and patient history to optimize treatment effectiveness in intensive care settings.

**Disclosure of Interest:**

None Declared

